# Identification of novel monocistronic HTLV-1 mRNAs encoding functional Rex isoforms

**DOI:** 10.1186/1742-4690-12-S1-P91

**Published:** 2015-08-28

**Authors:** Francesca Rende, Ilaria Cavallari, Vibeke Andresen, Valerio W Valeri, Donna M D'Agostino, Genoveffa Franchini, Vincenzo Ciminale

**Affiliations:** 1 Department of Surgery, Oncology and Gastroenterology, University of Padova, Padova, Italy; 2 Animal Models and Retroviral Vaccines Section, National Cancer Institute, Bethesda, MD, USA; 3 Department of Biomedical Sciences, University of Padova, Padova, Italy; 4 Istituto Oncologico Veneto-IRCCS, Padova, Italy; 5 current address, Centre of Cancer Biomarkers CCBIO, Translational Hemato-Oncology Group, Department of Clinical Science, University of Bergen, Norway; 6 current address, Novartis Vaccines Loc. Bellaria Rosia 53018 Sovicille (SI), Italy

## Background

HTLV-1 gene expression is controlled by the key regulatory proteins Tax and Rex. The concerted action of these proteins results in a two-phase kinetics of viral expression that depends on a time delay between their action. However, it is difficult to explain this delay, as Tax and Rex are produced from the same mRNA. In the present study we investigated whether HTLV-1 may produce novel mRNA species capable of expressing Rex and Tax independently.

## Findings

Results revealed the expression of 3 alternatively spliced transcripts coding for novel Rex isoforms in infected cell lines and in primary samples from infected patients. One mRNA coded for a Tax isoform and a Rex isoform, and 2 mRNAs coded for Rex isoforms but not Tax. Functional assays showed that these Rex isoforms exhibit activity comparable to canonic Rex. An analysis of the temporal expression of these transcripts upon ex vivo culture of cells from infected patients and cell lines transfected with a molecular clone of HTLV-1 revealed early expression of the dicistronic Tax/Rex mRNAs followed by the monocistronic mRNAs coding for Rex isoforms.

## Conclusions

The production of monocistronic HTLV-1 mRNAs encoding Rex isoforms with comparable activity to canonical Rex, but with distinct timing, would support a prolonged duration of Rex function with gradual loss of Tax, and would be consistent with the two-phase expression kinetics. A thorough understanding of these regulatory circuits will shed light on the basis of viral latency and provide groundwork to develop strategies for eradicating persistent infections.

